# Metanálisis sobre los niveles séricos de nesfatina-1 en la diabetes mellitus de tipo 2

**DOI:** 10.7705/biomedica.7708

**Published:** 2025-08-11

**Authors:** Elizabeth Reyes-Lucía, Angélica Ramírez-Guerrero, Christian González-Villaseñor, Nelly Macías-Gómez

**Affiliations:** 1 Laboratorio de Genética Humana, Centro Universitario del Sur, Ciudad Guzmán, Jalisco, México Centro Universitario del Sur Centro Universitario del Sur Ciudad Guzmán Jalisco México

**Keywords:** nucleobindinas, diabetes mellitus de tipo 2, biomarcadores, ensayo de inmunoadsorción enzimática, metaanálisis., Nucleobindins, diabetes mellitus, type 2, biomarkers, enzyme-linked immunosorbent assay, meta-analysis.

## Abstract

**Introducción.:**

La nesfatina-1 es la proteína codificada por el gen *NUCB2* y que, recientemente, se ha asociado con la síntesis y la regulación de la insulina, la homeostasis de la glucosa y su posible participación en la etiopatogenia de la diabetes mellitus de tipo 2.

**Objetivo.:**

Analizar la relación entre los niveles séricos de nesfatina-1 y la diabetes mellitus de tipo 2.

**Materiales y métodos.:**

Se incluyeron artículos de las plataformas PubMed, Scopus y DOAJ, publicados entre el 2012 y el 2024. Las palabras clave de la búsqueda fueron “diabetes mellitus”, “diabetes”, “diabetes mellitus tipo 2”, “nesfatin-1”, “*NUCB2*”, “ELISA”, “plasma” y “suero”. Se eligieron estudios de casos y controles y de diseño transversal realizados en humanos, que tuvieran acceso al texto completo y contaran con la cuantificación de nesfatina-1.

**Resultados.:**

Para el análisis estadístico, se incluyeron ocho artículos con un total de 305 pacientes con diabetes mellitus de tipo 2 y 205 controles. Los resultados mostraron una relación significativa entre los valores de nesfatina-1 y el desarrollo de diabetes mellitus de tipo 2, con un alto índice de heterogeneidad entre los estudios (t^2^= 3,91; x^2 =^ 349,63, p < 0,00001; I^2^= 98%).

**Conclusiones.:**

Los resultados muestran una relación significativa entre las concentraciones de nesfatina-1 y la diabetes mellitus de tipo 2, lo que respalda su utilidad como biomarcador de esta enfermedad.

## Abstract.

**Introduction.:**

Nesfatin-1, a peptide coded by the *NUCB2* gene, has recently been implicated in the regulation of insulin synthesis and glucose homeostasis. These findings suggest a potential role for nesfatin-1 in the etiology of type 2 diabetes mellitus.

**Objective.:**

To analyze the relationship between circulating levels of nesfatin-1 and type 2 diabetes mellitus.

**Materials and methods.:**

We used PubMed, Scopus, and DOAJ platforms to search published articles from 2012 to 2024. The keywords used were “diabetes mellitus”, “diabetes”, “type 2 diabetes”, “nesfatin-1”, “*NUCB2*”, “ELISA”, “plasma”, and “serum”. We included all case-control and cross-sectional studies conducted in humans, with full-text availability, and written in English or Spanish.

**Results.:**

We included eight studies comprising 305 patients with type 2 diabetes mellitus and 205 controls. The results showed a significant relationship between nesfatina-1 and type 2 diabetes mellitus with a high heterogeneity index (t^2^ = 3.91; χ^2^ = 349.63, p < 0.00001; I^2^ = 98%).

**Conclusions.:**

The results show a significant relationship between nesfatin-1 and type 2 diabetes mellitus, supporting its potential use as a biomarker for this disease.

De acuerdo con la Organización Mundial de la Salud (OMS), la diabetes mellitus se describe como una enfermedad metabólica crónica caracterizada por hiperglucemia, capaz de ocasionar complicaciones cardiacas, vasculares, oculares, renales y del sistema nervioso periférico ^1^.

La Federación Internacional de Diabetes estima que 537 millones de adultos entre los 20 y los 79 años padecen de diabetes. Se pronostica que este número aumente a 643 millones en el 2030 y a 783 millones en el 2045. La diabetes mellitus de tipo 2 es la más común, ya que representa más del 90 % de los casos de diabetes en todo el mundo ^2^.

La diabetes mellitus de tipo 2 es una enfermedad crónica, degenerativa, multifactorial y poligénica. Sus factores de riesgo incluyen variables modificables y no modificables, como la edad, el origen étnico, el estilo de vida y la obesidad abdominal ^3^. A medida que se incrementa la edad, también lo hace la probabilidad de que se desarrolle diabetes mellitus de tipo 2, debido a la disminución de la masa muscular magra y la funcionalidad de las células (3 del páncreas. El origen étnico es otro factor determinante, influenciado por la interacción de factores ambientales, culturales, socioeconómicos y dietéticos. El estilo de vida con poca actividad física y una dieta rica en grasas saturadas y azúcares refinados, consumo de alcohol y tabaco, también implica mayor riesgo ^4^. La obesidad abdominal (diagnosticada fácilmente con la determinación del perímetro de la cintura) representa un riesgo 1,63 veces mayor de desarrollar diabetes mellitus en hombres con un perímetro de más de 90 cm, y mayor de 2,82 veces, en mujeres con un perímetro de más de 80 cm ^5^.

Los factores genéticos son una de las principales variables no modificables implicadas en el desarrollo de la diabetes mellitus de tipo 2. Se han identificado aproximadamente 40 variantes genéticas que pueden aumentar hasta 2,5 veces la probabilidad de desarrollar diabetes respecto a aquellos que no las portan.

Recientemente, se ha propuesto la proteína nesfatina-1 -codificada por el gen *NUCB2*- como un marcador para el desarrollo de diabetes mellitus de tipo 2; sin embargo, los resultados aún son contradictorios. El gen *NUCB2* se encuentra en el brazo corto del cromosoma 11, específicamente en el locus 11p15.1, y codifica para una proteína de 420 aminoácidos (aa), de los cuales 24 corresponden al péptido de señalización y 396 a la forma precursora. La proproteína es procesada por la prohormona convertasa 1/3 (PC1/3), lo que da lugar a los fragmentos nesfatina-1 (1-82 aa), nesfatina-2 (85-163 aa) y nesfatina-3 (166-396 aa) ^6^.

La nesfatina-1 presenta tres dominios: el N-terminal (N23), la secuencia central (M30) y el C-terminal (C29). El dominio M30 está asociado con la actividad biológica del péptido ^7^. Este fragmento, descubierto en el 2006, es capaz de atravesar la barrera hematoencefálica ^8^ y se ha detectado en el sistema nervioso central y en diversos tejidos, como las reservas de tejido adiposo en todo el organismo, las células β del páncreas y la mucosa gástrica, entre otros ^9^. La nesfatina-1 aún es un ligando huérfano porque no se ha identificado su receptor. No obstante, se ha sugerido que podría interactuar con el receptor de la grelina, dado que las dos moléculas coexisten en las células de tipo X/A de las glándulas oxínticas del fondo gástrico. Allí, los dos péptidos son excretados como prohormonas y convertidos finalmente en grelina y nesfatina-1 ^10^^,^^11^.

La nesfatina-1 desempeña diversas funciones; entre las más importantes, está mantener el equilibrio energético mediante la homeostasis de los niveles de glucosa en la sangre y regular la temperatura corporal ^12^. En el sistema nervioso central, la nesfatina-1 regula la ingestión de alimentos y está clasificada como una hormona anorexigénica que juega un importante papel en los procesos de la obesidad ^13^.

En estudios *in vitro*, se ha demostrado que estimula la producción de insulina según los niveles de glucosa en sangre, mediante la activación de los canales de calcio de tipo L, e inhibe los canales de potasio en las células β de los islotes pancreáticos ^14^. Además, se ha documentado que la nesfatina-1 tiene un efecto hipoglucemiante en los tejidos periféricos al estimular la fosforilación de AKT y promover la expresión del transportador de glucosa 4 (GLUT4), principalmente en el músculo esquelético y el tejido adiposo. Asimismo, la nesfatina-1 actúa como una adipocina, lo que sugiere un posible vínculo mecánico entre la resistencia a la insulina y la diabetes mellitus de tipo 2.

La nesfatina-1 modula el metabolismo de la glucosa mediante la fosforilación de proteínas de señalización -como la proteína cinasa activada por AMP-y aumenta la sensibilidad del hígado a la insulina, todo lo cual ayuda a regular el apetito y la grasa corporal ^15^. En los estudios realizados en pacientes con diferentes trastornos del metabolismo de la glucosa (intolerancia a la glucosa, síndrome metabólico y diabetes mellitus de tipo 2), se ha reportado disminución de los niveles de nesfatina-1, la cual fue más evidente en aquellos con diabetes mellitus de tipo 2, por lo que se ha sugerido dicho péptido como un marcador de evolución de la enfermedad ^16^. Algunas investigaciones reportan una asociación fuerte entre la variante rs11024251 de *NUCB2* y la diabetes mellitus de tipo 2 (p = 2,97x10"^6^); además, las variantes rs10832757 y rs11024251 se vincularon con su desarrollo en los hombres (p = 0,0244), y las variantes rs1330, rs10766383, rs10832757 y rs11024251, en las mujeres (p < 0,05) ^17^.

Hasta el momento, hay controversia respecto a los niveles de nesfatina-1 en los pacientes con diabetes mellitus de tipo 2, ya que en algunos estudios dichas concentraciones se encontraron elevadas, mientras que, en otros, se encontraron disminuidas ^18^.

En el presente trabajo, se llevó a cabo un metanálisis de estudios transversales y de casos y controles, en los cuales se evaluaron los valores séricos de nesfatina-1 en casos de diabetes mellitus de tipo 2, publicados entre el 2012 y el 2024.

## Materiales y métodos

La presente revisión sistemática y el metanálisis se realizaron de acuerdo con la declaración PRISMA (*Preferred Reporting Items for Systematic Reviews and Meta-Analyses*) del 2020 ^19^.

### Método de búsqueda

La búsqueda de los artículos científicos fue hecha de forma independiente por cuatro investigadores, entre enero y marzo del 2024. Se utilizaron las plataformas PubMed, Scopus y DOAJ (*Directory of Open Access Journals*). Para la búsqueda, se usaron los siguientes términos MeSH (*Medical Subject Headings*): “nesfatin-1” (ID: C000630650), “*NUCB2*” (ID: C000630650), “diabetes mellitus tipo 2” (ID: D003924), “ELISA” (ID: D004797), “serum” (ID: D044967) y “plasma” (ID: D010949), unidos por los conectores *AND* y *OR*.

Para la selección de los artículos científicos, se realizó una primera selección basada en el título, el resumen y la disponibilidad de acceso libre o del texto completo para su lectura y análisis. Las referencias citadas en los artículos incluidos se revisaron con el objetivo de detectar las de mayor relevancia para el presente análisis.

### Criterios de inclusión y exclusión

Los criterios de inclusión de los artículos contemplaron:

1. estudios realizados en seres humanos;
2. mención del consentimiento informado basado en la Declaración de Helsinki;
3. diseño metodológico transversal o de casos y controles;
4. publicación entre el 2012 y el 2024;
5. detección de nesfatina-1 mediante la técnica ELISA, y
6. artículos redactados en español o inglés.

Los criterios de exclusión fueron:

1. determinación de nesfatina-1 en muestras diferentes al plasma o suero;
2. estudios en seres humanos con diagnóstico distinto a diabetes mellitus de tipo 2, y
3. estudios con información incompleta para la realización de los análisis.

### Extracción de datos

Una vez seleccionados los artículos, se creó una base de datos en el programa Excel®, en la cual se detallaron: 1) autor principal y colaboradores; 2) nombre completo del artículo; 3) año de la publicación; 4) nombre de la revista; 5) idioma; 6) tipo de población y país de origen de los individuos; 7) tipo de estudio; 8) variables incluidas en el análisis; 9) edad de los participantes; 10) método de diagnóstico y clasificación de la diabetes mellitus de tipo 2; 11) resultado de los niveles de nesfatina-1 en suero; 12) criterios de selección; 13) aspectos éticos del estudio, y 14) conclusiones.

### Evaluación de la calidad de los estudios

La calidad de los estudios seleccionados se analizó utilizando la escala Newcastle-Ottawa (NOS) para los de casos y controles; un reporte con una puntuación de 7 o menos, se consideró de alta calidad ^20^ (cuadro 1). Para los artículos con diseño experimental de tipo transversal, se utilizó la escala de la *Agency for Healthcare Research and Quality* (ARHQ); se incluyeron aquellos con una puntuación de 7 (moderada calidad) y 9 (alta calidad) ^21^ (cuadro 2).

**Cuadro 1.** Escala de Newcastle-Ottawa para estudios de casos y controles. La escala comprende tres dimensiones: selección, comparabilidad y exposición. Cada dimensión tiene diferentes criterios que se califican mediante un sistema de estrellas. Se otorga un máximo de una estrella a cada criterio, con excepción del dominio de comparabilidad, cuyo máximo son dos estrellas. La máxima calificación para un estudio de alta calidad es de nueve puntos.

Criterios	**Khalil *et al.***	**Mirakhor *et al.***	**Kadim *et al*.**
	^29^	^27^	^15^
Selección			
1 ¿Es adecuada la definición de caso?	★	★	★
2 Representatividad de los casos	★	★	★
3 Selección de controles	★	★	★
4 Definición de controles	★	★	★
Comparabilidad			
5 Comparabilidad de casos y controles sobre la base	★ ★	★ ★	★ ★
del diseño o del análisis			
Exposición			
6 Comprobación de la exposición	-	-	-
7 Mismo método de verificación para casos y controles	★	★	★
8 Tasa sin respuesta	-	-	-
Total	7	7	7

Disponible en: https://www.ohri.ca/programs/clinical_epidemiology/oxford.asp

**Cuadro 2.** Lista de verificación de la metodología Agency for Healthcare Research and Quality, compuesta de 11 criterios. Cada criterio se califica con un puntaje de 0 a 1, para un total máximo de 11 puntos.

Criterios	**Zhang *et al*.**^32^	**Matta *et al.*** ^28^	**Abed *et al.*** ^31^	**Huang *et al.*** ^30^	**Algul *et al*.**^16^
1 Define fuente de información (encuesta y revisión de registros).	1	1	1	1	1
2 Enumera los criterios de inclusión y exclusión de sujetos expuestos y no expuestos (casos y controles) o consulta publicaciones anteriores.	1	1	0	1	1
3 Indica el período utilizado para identificar pacientes.	0	1	1	1	0
4 Indica si los sujetos fueron consecutivos o no, o si no se basaron en la población.	1	1	1	1	1
5 Indica si los evaluadores de los componentes subjetivos del estudio estaban cegados a otros aspectos del estado de los participantes.	1	1	1	1	1
6 Describe cualquier evaluación realizada para propósitos de aseguramiento de la calidad (por ejemplo, prueba de mediciones de resultados primarios).	1	1	1	1	1
7 Explica cualquier exclusión de pacientes del análisis.	1	1	0	1	1
8 Describe cómo se evaluaron o controlaron los factores de confusión.	1	1	1	1	1
9 Explica cómo se manejaron los datos faltantes en el análisis.	0	0	0	0	1
10 Resume las tasas de respuesta de los pacientes y la integridad de la recopilación de datos.	1	1	1	1	1
11 Aclara qué seguimiento, si lo hubiera, se esperaba y el porcentaje de pacientes para los cuales se obtuvieron datos o seguimientos incompletos.	0	0	0	0	0
Total	8	9	7	9	9

### Análisis estadístico

La relación de los niveles de nesfatina-1 entre controles y pacientes con diabetes mellitus de tipo 2 se evaluó con la prueba de X^2^, con un intervalo de confianza (1C) del 95 %; un valor de p ≤ 0,05 se consideró significativo. Para identificar el grado de heterogeneidad entre los estudios incluidos, se utilizó la prueba Q de Cochran, y se calculó el valor de I^2^ y de t^2^. Se consideraron significativos un valor de t^2^ menor de 0,05 y uno de I^2^ mayor del 50 %.

Para este análisis, se empleó el *software Cochrane Review Manager*, versión 5.4 (RevMan) ^22^. Para la evaluación del sesgo de los artículos seleccionados en el metanálisis, se aplicaron las pruebas de Begg y Egger (Epidat, versión 3.1) ^23^. Se consideró que un valor de p ≥ 0,05 indicaba ausencia de sesgo.

## Resultados

**Figura 1.**
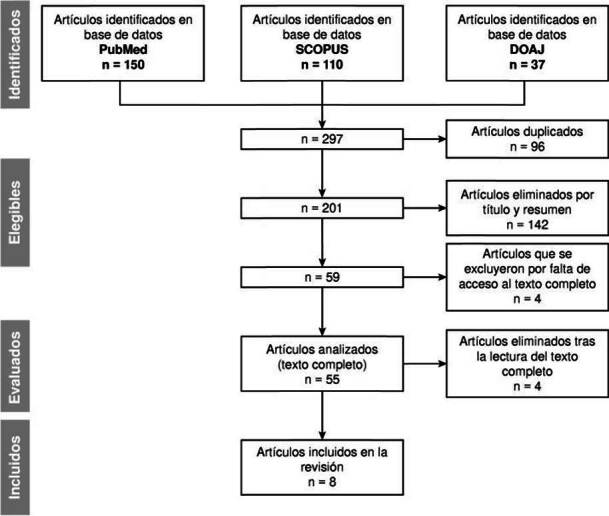
Diagrama de flujo de la metodología utilizada en la selección de los artículos del metanálisis.

EI resultado de la búsqueda en las diferentes plataformas arrojo un total de 297 artículos. Se excluyeron 96 reportes duplicados, 142 tras la lectura del resumen y 4 por falta de acceso al texto completo, lo que redujo el número de artículos a 54. Tras la lectura completa de los 54 artículos, se eliminaron 46 por no cumplir con los criterios de inclusión y se seleccionaron 8 para este metanálisis (figura 1).

### Características de los estudios incluidos

Respecto al origen de los estudios incluidos en este metanálisis, dos se realizaron en una población de Irak, uno en Turquía, uno en Irán, uno en Egipto y dos en China. Todos ellos se encontraban escritos en el idioma inglés. Tres estudios tenían un diseño de casos y controles, mientras que los cinco restantes eran estudios transversales.

En relación con el diagnóstico de diabetes mellitus de tipo 2 de los casos incluidos en los estudios, dos de ellos se basaron en los criterios establecidos por la Organización Mundial de la Salud (OMS), cuatro en los de la *American Diabetes Association* (ADA) y en dos estudios no se especificaron los criterios diagnósticos (cuadro 3).

**Cuadro 3.** Características de los estudios incluidos en el metanálisis. Los datos se presentan como media más o menos la desviación estándar.

Estudio	Año	País	Criterios DM2	Tipo de muestra	Método	Edad (años)		Tamaño de muestra (H/M)		Nesfatina-1 (ng/ml)	
						Casos	Controles	Casos	Controles	Casos	Controles
Zhang *et al*. ^32^	2012	China	OMS	Plasma	ELISA	54 ± 11	51 ±7	74 (39/35)	73 (36/37)	1,91 ±0,79	1,41 ±0,58
Algul *et al*. ^16^	2016	Turquía	ADA	Suero	ELISA	40,6 ± 1,4	39,2 ± 1,2	20 (SD/SD)	20 (SD/SD)	0.867 ± 0,02	1,094 ±0,07
Kadim *et al*. ^15^	2022	Irak	-	Suero	ELISA	54,23 ±2,13	48,18 ±3,60	30(18/12)	30(18/12)	51,26 ± 7,05	82,53 ±13,16
Mirakhor *et al*. ^27^	2019	Irán	OMS	Suero	ELISA	44,97 ± 10,05	43,43 ± 10,45	30 (14/16)	30(17/13)	0,99 ± 0,29	2,61 ± 0,92
Matta *et al*. ^28^	2022	Egipto	ADA	Suero	ELISA	48,7 ±8,8	48,16 ± 10,6	30(17/13)	28(15/13)	0,0389 ±0,011	0,154 ±0,035
Khalil *et al*. ^29^	2024	Egipto	ADA	Suero	ELISA	51,88 ±9,27	48,90 ±8,15	60 (27/33)	30 (13/17)	5,07 ± 1,78 mmol/L	9,05 ± 2,1 mmol
Abed *et al*. ^31^	2023	Irak	-	Suero	ELISA	48,31 ± 8,42	48.41 ± 8,45	60 (31/29)	30 (17/13)	4,96 ± 1,03	1,71 ±0,80
Huang *et al*. ^30^	2022	China	ADA	Suero	ELISA	54 ±2,5	52 ±5	75 (41/34)	37 (23/14)	0,623 ±0,218	1,060 ± 0,823

DM2: diabetes mellitus de tipo 2; H/M: hombres/mujeres; QMS: Organización Mundial de la Salud; ADA: American Diabetes Association: SD: sin dato

### Análisis general

**Figura 2.**
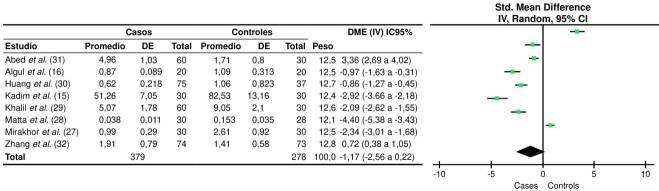
Gráfico de bosque de los niveles de nesfatina-1 en pacientes con diabetes mellitus de tipo 2 realizado con el programa RevMan Cochrane, versión 5.4. Se muestra la diferencia de medias estandarizada mediante varianza invertida, método aleatorio e intervalo de confianza del 95 %. DE: desviación estándar; DME: diferencia de medias estandarizada; IV: varianza invertida; 1C: intervalo de confianza Heterogeneidad: i^2^ = 3,91; x^2^ = 349,63, df = 7 (p < 1 x 10'^5^); I^2^ = 98 % (p < 0.00001) Prueba deefecto global: Z = 1,65 (p = 0,10)

En el presente análisis, se demuestra una estrecha asociación entre los niveles de nesfatina-1 y la diabetes mellitus de tipo 2, con una diferencia estadísticamente significativa de p = 0,00001. Los grupos analizados presentaron una gran heterogeneidad, con un I^2^ = 98 % y un t^2^ = 3,91, de acuerdo con la prueba Q de Cochran. El sesgo de los artículos publicados en el metanálisis, se consideró insignificante (prueba de Begg: p = 0,1078; prueba de Egger: p = 0,2153) (figura 2).

## Discusión

La diabetes mellitus de tipo 2 es una enfermedad crónica, degenerativa, muy frecuente a nivel mundial; México, por ejemplo, ocupa el séptimo lugar en número de casos ^2^. Sin duda alguna, esta enfermedad es una de las más discapacitantes en México, ya que, entre aquellos que la presentan, 6,2 millones padecen insuficiencia renal en distintos estadios de evolución ^24^ con una tasa de amputación de extremidades inferiores de 9,2 por cada 100.000 habitantes sanos ^25^ y una mortalidad atribuible del 14 % ^26^.

En años recientes, se ha relacionado al neuropéptido nesfatina-1 con su desarrollo; sin embargo, los resultados han sido controversiales. En el presente metanálisis, se incluyeron ocho artículos publicados entre el 2012 y el 2024. Estos estudios muestran una asociación significativa entre la disminución de los niveles de nesfatina-1 y la diabetes mellitus de tipo 2 (26-32). Es importante destacar que los estudios aquí analizados también incluyen información relevante para determinar el papel de dichas concentraciones en la población afectada.

En uno de los estudios incluidos, el de Kadim *et al*. ^15^, se compararon pacientes con diabetes mellitus de tipo 2 recién diagnosticada con pacientes con un curso prolongado de la enfermedad. Estos investigadores observaron también que los niveles de nesfatina-1 eran menores en los pacientes con diabetes mellitus de tipo 2 (58,31 ng/ml) respecto a los del grupo control (82,53 ng/ml; p < 0,05). Al comparar el grupo de pacientes con diagnóstico reciente con aquellos con un curso crónico, observaron que, en este último grupo, los niveles de nesfatina-1 eran aún más bajos (51,26 ng/ml) que en los primeros (67,08 ng/ml; p < 0,05). Asimismo, se observó una correlación negativa entre los niveles de nesfatina-1, la concentración y la resistencia a la insulina (esta última calculada según el modelo HOMA-IR - *Homeostatic Model Assessment for Insulin Resistance*). Por esta razón, la nesfatina-1 se ha sugerido como un marcador de evolución de la diabetes mellitus de tipo 2 ^15^.

Es importante destacar que en el estudio reportado por Algul *et al.*^16^, además de aquellos con diabetes mellitus de tipo 2 -que mostraron niveles bajos de nesfatina-1 en comparación con los controles (0,867 ng/ml; p = 0,007)-, se incluyeron pacientes con intolerancia a la glucosa y síndrome metabólico, los cuales tuvieron bajas concentraciones de nesfatina-1 (1,039 ng/ml; p = 0,5 y 0,885; p = 0,01). En el presente estudio, se observa cómo disminuyen los valores séricos de nesfatina-1 en las diferentes etapas de la diabetes mellitus de tipo 2, con una reducción significativa a partir de la manifestación del síndrome metabólico ^16^. Lo anterior refuerza la propuesta de considerar a la nesfatina-1 como un marcador de la evolución de esta enfermedad.

Una limitación del presente metanálisis es que en los estudios analizados no se informa sobre la evolución de los pacientes con la enfermedad. Este punto sería de vital relevancia para el análisis, ya que otros autores mencionan que los valores séricos de nesfatina-1 pueden variar, dependiendo de si se administra tratamiento o no se hace. El conocer esta información podría ofrecer una mejor perspectiva sobre la utilidad de la proteína cuantificada como indicador pronóstico de la evolución de la enfermedad.

Por otro lado, aunque algunos estudios aportaban información importante sobre el tema, no pudieron incluirse en este metanálisis por haber sido llevados a cabo en modelos animales o *in vitro*.

El presente metanálisis mostró una asociación significativa entre las concentraciones de nesfatina-1 y la presencia de diabetes mellitus de tipo 2, por lo que dicho péptido podría utilizarse como biomarcador en el diagnóstico de este padecimiento.

Finalmente, consideramos que es de vital importancia hacer estudios familiares en casos de diabetes mellitus de tipo 2, una enfermedad compleja multifactorial, pues se podrían establecer los puntos de corte de los valores séricos de la nesfatina-1, para la prevención, el diagnóstico y la evolución de la enfermedad.
